# GluN3A: An NMDA Receptor Subunit with Exquisite Properties and Functions

**DOI:** 10.1155/2013/145387

**Published:** 2013-12-09

**Authors:** Laura A. Kehoe, Yann Bernardinelli, Dominique Muller

**Affiliations:** ^1^Department of Neuroscience, University of Geneva, CMU, 1211 Geneve 4, Switzerland; ^2^Cellular Neurobiology, Departamento de Neurociencias, Centro de Investigacion Médica Aplicada (CIMA), Universidad de Navarra, 31008 Pamplona, Spain

## Abstract

N-methyl-D-aspartate receptors (NMDAR) are pivotal for synaptic plasticity and memory formation. Conventional NMDAR consist of heterotetrameric structures composed of GluN1 and GluN2 subunits. A third subunit, GluN3, can also assemble with NMDAR subunits giving a remarkable modification of their heteromeric structure, forming a “nonconventional” NMDAR. As a consequence, the stoichiometry and kinetic properties of the receptors are dramatically changed. Among the GluN3 family, the GluN3A subunit has been the focus of a large amount of studies during recent years. These studies reveal that GluN3A is transiently expressed during development and could play a role in the fine tuning of neuronal networks as well as associated diseases. Moreover, GluN3A distribution outside the postsynaptic densities, including perisynaptic astrocytes, places it at a strategic position to play an important role in the interactions between neurons and glial cells. This review highlights GluN3A properties and addresses its role in neurophysiology and associated pathologies.

## 1. Introduction

The glutamatergic network during postnatal development is under a tight regulation controlled by activity. This activity is mediated by postsynaptic ionotropic glutamate receptors (iGluR), NMDAR, and *α*-amino-3-hydroxy-5-methyl-4-isoxazole propionate receptors (AMPAR) as well as metabotropic glutamate receptors (mGluR) [1]. Indeed, activation of NMDAR promotes the insertion of AMPAR to the synapse, inducing long-term potentiation (LTP) [2]. In contrast, a reduction in NMDAR activation promotes the removal of AMPAR, provoking long-term depression (LTD) [3]. These functional synaptic plasticity properties are tightly linked with structural modifications such as enlargements and reductions in dendritic spine size or even formation and elimination of synapses [4–6]. These mechanisms are directly influenced by postsynaptic calcium (Ca^2+^) [7], and Ca^2+^ influx is strongly controlled by NMDAR subunit composition [8, 9]. While GluN1 and GluN2 are the main subunits forming functional NMDAR [10–12], a third member of the family, GluN3, provides entirely new properties to NMDAR kinetics, especially with regard to Ca^2+^ permeability [13, 14]. When coassembled with GluN1 and GluN2 subunits, GluN3A exerts a dominant-negative effect on NMDAR properties [13, 15, 16]. Its presence dominates the properties of NMDAR resulting in a negative action on NMDAR, that is, insensitivity to magnesium (Mg^2+^) and reduced Ca^2+^ influx. Predominately expressed during post-natal development, GluN3A has a strong impact on dendritic spine densities [17, 18] and is consequently a key player in controlling glutamatergic synaptic development and plasticity [19].

Recent advances in understanding glutamatergic synapse function and structure have also revealed astrocytes to be an active component of the synapse [20, 21]. Astrocytes participate in the modulation of synaptic transmission as well as in LTP through the release of glutamate and D-Serine in a Ca^2+^-dependent manner [22, 23]. These gliotransmitters can act on neuronal GluN3-containing NMDAR located at extrasynaptic sites [22, 24]. Moreover, astrocytes also express GluN3-containing NMDAR [25] that could influence their intracellular Ca^2+^ homeostasis. Thus GluN3 could be crucial for the bidirectional communication between astrocytes and neurons.

This review provides an up-to-date overview of recent findings on GluN3A. Moreover, particular focus will be made on its role in synapse dynamics, disease, and interactions between neurons and astrocytes.

## 2. Conventional NMDAR Channel Properties

NMDAR are comprised of seven subunits divided into three main subfamilies: the obligatory GluN1 subunit, four GluN2 subunits (A-D), and two GluN3 subunits (A-B). GluN1 and GluN2-containing receptors are the most abundant NMDAR complexes throughout the central nervous system (CNS). The GluN2 subunits determine channel properties and the subcellular localization of the receptor. NMDAR have characteristic properties unlike that of AMPAR and kainate receptor family, exhibiting a strong voltage-dependent block by Mg^2+^, high permeability to Ca^2+^, and slow gating kinetics. However, these properties can vary dramatically depending on the expression and the composition of the subunits within the receptor.

The activation of NMDAR requires the coordinated binding of glycine and glutamate to the ligand binding domain (LBS) site on GluN1 and GluN2 subunits, respectively. This triggers the conformation change of the receptor, allowing the flow of ions and depolarization of the postsynaptic site [26]. GluN2A-containing receptors display faster kinetics, a higher open probability, and faster deactivation kinetics compared with GluN2B-containing receptors [26, 27]. In addition, GluN2C-containing NMDAR exhibit unique properties of low conductance, low open probability, and low sensitivity to Mg^2+^ [28–30]. Furthermore, the presence of GluN2D-containing NMDAR leads to extremely slow decay times [26, 31]. In contrast, GluN3A-containing NMDAR bring about distinct nonconventional properties to the NMDAR complex.

## 3. GluN3A “Nonconventional” Properties

Since its discovery in 1995 by two independent groups [15, 16], GluN3A (then termed Chi-1 and NMDAR-L) has sparked great interest due to its particular properties within the NMDAR family. Although it shares a low sequence homology with GluN1 and GluN2 (~27%), it holds specific characteristics of the NMDAR family [15, 16]. Such features consist of (i) a large N-terminal extracellular domain with multiple glycosylation sites, (ii) three transmembrane domains (M1, M3 and M4), with M2 being a reentrant loop, (iii) a hydrophobic sequence just upstream of M1 within the N-terminal domain (NTD) known as the S1 region giving rise to the LBD, (iv) a large extracellular domain between M3 and M4 housing the S2 region, and (v) a unique C-terminal domain (CTD) of GluN3A that holds specific glycosylation and phosphorylation sites, differing from GluN1 and GluN2 subunits [15, 16]. More specifically, the M2 region has been described as controlling the ion selectivity of the glutamate-gated channels [32] due to the presence of a QNR site (glutamine(Q)/asparagine(N)/arginine(R)). In the case of GluN3A the presence of an arginine is adjacent to this site [15, 16]. Site directed mutagenesis indicates that the QNR site influences the flow of divalent ions, specifically controlling the permeability of Ca^2+^, and influencing the Mg^2+^ block of recombinant NMDAR.

As with all other subunits of NMDAR, GluN3A also exhibits a bilobed extracellular domain, formed by the S1 region of the NTD and the S2 segment of the extracellular loop between M3 and M4 domains. This S1S2 segment forms the LBD for all subunits [33]. Both GluN1 and GluN3 subunits bind the coagonist glycine for NMDAR activation. GluN3A binds glycine at a much higher affinity than GluN1, almost 650 times better than GluN1 [33], which gives a unique profile that is selectively different from GluN1. In addition to glycine, D-serine also acts as an agonist at GluN3A subunits, again with a higher affinity than GluN1.

When GluN1 and GluN2 subunits coassemble, they form a core which regulates the channels permeation to ions [34–37]. When GluN3A is coexpressed with GluN1, this channel permeation pathway is also formed. The alignment of a ring of polar threonine residues in both GluN1/3A assemblies forms a constriction in the outer vestibule of the channel. This disturbs the chain of Ca^2+^ binding sites that usually facilitate the Ca^2+^ flux, thus reducing Ca^2+^ permeability [37], a characteristic of GluN3A-containing NMDAR.

### 3.1. Triheteromeric NMDAR Containing GluN3A

**Figure 1:**
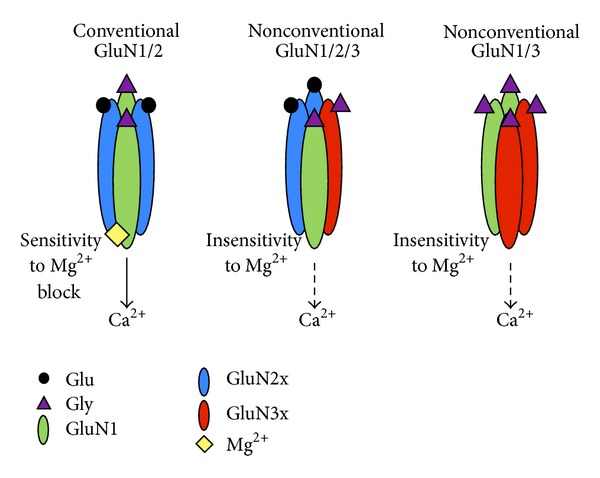
Schematic illustrating the conventional NMDAR containing GluN1 and GluN2 subunits (left), a nonconventional heterotrimeric NMDAR containing all three subunits (middle), and a glycine diheteromeric NMDAR containing GluN1 and GluN3 subunits (right). The main properties that are changed in these nonconventional NMDAR are the agonist binding, the insensitivity to Mg^2+^, and the low permeability to Ca^2+^. Thus GluN3-containing NMDAR exert a dominant-negative effect on NMDAR properties. Glu: glutamate, Gly: Glycine, Mg^2+^: magnesium, and Ca^2+^: calcium.

The distinct properties of NMDAR comprised of GluN1, GluN2, and GluN3A subunits have been consistently reported in various recombinant and transgenic systems [13, 38]. GluN3A forms stable biochemical complexes with the other NMDAR subunits [38]. NMDAR comprised of GluN1/2A or 2B in both low and physiological extracellular Ca^2+^ conditions induce just one large conductance state. The presence of GluN3A results in two distinct independent conductance states: the typical large conductance state, similar to what is found in conventional receptors, and the second significantly smaller, which exhibits a slight increase in mean opening time [14, 17, 38]. Ca^2+^ permeability in GluN3A-containing NMDAR is significantly reduced [13, 14, 38] due to the constriction of the outer vestibule [37]. Another prominent feature of GluN3A-containing receptors is the insensitivity to Mg^2+^ block at hyperpolarized potential: even with varying concentrations of Mg^2+^, GluN3A prevents a Mg^2+^ block [13, 38] (Figure 1); these properties are further characterized in other reviews on GluN3; see [39–41]. Overall, these properties promote a reduction in NMDA-induced currents. In contrast, the amplitudes of NMDA-induced currents in GluN3A KO neurons are larger in cerebral cortical neurons compared to WT neurons [17]. However, this is only detected during development as in adulthood the levels reach normal values.

Functional NMDAR require the combination of one or two GluN1 subunits with either one or two of the GluN2 subunits; and/or a combination of GluN3 subunits [42, 43]. GluN1 is the obligatory subunit which is always present in functional NMDAR complexes [12, 29]. The tetrameric structure of NMDAR was suggested to be arranged in a couple of dimers in a 1-1-2-2 orientation (i.e., GluN1-GluN1 and GluN2-GluN2) heavily dependent on the final transmembrane domain and the CTD [44]. However, evidence now suggests that the GluN1 subunits are arranged in dimers and the GluN2 subunits are simply added to form the tetramer [45]. It still remains unclear how the addition of the GluN3 subunit fits into this stoichiometric formation. Although GluN3A can form functional NMDAR complexes with GluN1 and GluN2 subunits [46], it has been proposed that neither GluN2 or GluN3A subunits can form homo-oligomers, unlike the GluN1 subunit [47]. Evidence proposes a two step assembly process for NMDAR containing GluN3A; first a GluN1 subunit associates with either a GluN2 or GluN3 subunit into heterodimers. Secondly, these two heterodimers form the final tetrameric subunit arrangement [47]. The precise stoichiometry remains largely unknown, and it could differ in brain region, cell type and during development. In addition to the heterotrimeric glutamate receptors, evidence suggests a possible excitatory glycine receptor in the form of a diheteromeric GluN1/3 receptor.

### 3.2. Diheteromeric NMDAR Containing GluN3A

Interestingly, GluN1 can coassemble with either GluN3A and/or GluN3B and form functional excitatory glycine receptors [46, 48]. These NMDAR are not activated by glutamate. However, activation of these receptors by glycine leads to extremely low permeability to Ca^2+^, low sensitivity to Mg^2+^, and triggers bursts of firing [49, 50]. As both the GluN1 and GluN3 subunits have a glycine binding site, it would be expected that binding of glycine to both subunits is sufficient for receptor activation. However, coapplication of Zinc (Zn^2+^), which usually acts on GluN2 subunits [51], may also act as both a potent positive modulator and an agonist at these GluN1/3 NMDAR [52]. The full extent of expression of these GluN1/3A glycine excitatory receptors *in vivo* remains largely unknown and very little literature has focused on the impact of the GluN3B subunit on NMDAR function. Two possibilities proposed in a review by Pachernegg et al., [40] suggested that glycine could be saturated at these receptors and provoke a more depolarized state, implicating action potentials and synaptic transmission. Furthermore, their effects presynaptically could reduce firing frequency and postsynaptically induce an increase in evoked potentials [40]. Further studies are needed to elucidate a role of these GluN1/3 receptors *in vivo*.

### 3.3. Membrane Targeting of GluN3A

Correct assembly and trafficking of the NMDAR complex are critical for functional surface expression. As with GluN2 subunits, GluN3A subunits are dependent on the coassembly with GluN1 subunits to be expressed at the surface membrane [14, 53]. Furthermore, GluN2 subunits contain a PDZ domain in their CTD that interacts with PSD95/SAP102 scaffolding proteins [26] influencing NMDAR anchoring and stabilization within the postsynaptic density. To date it appears that like GluN1 [54], GluN3A does not contain a PDZ domain and therefore would require the coassembly with GluN2 subunits to be targeted to the postsynaptic density (PSD).

GluN3A and GluN1-1a subunits are assembled at early stages in the biosynthetic pathway at the level of the endoplasmic reticulum (ER). Only when GluN3A is coassembled with GluN1-1a can it then exit the ER and be trafficked to the surface. In the absence of GluN1-1a, GluN2 and GluN3 are retained in the ER, strongly suggesting that GluN1-1a is necessary for receptor trafficking and efficient membrane insertion [14].

All NMDAR subunits contain some form of an ER retention signal in their CTD. It could be speculated that the correct folding and coassembly of the subunits mask the ER retention signals allowing for GluN1 to guide the trafficking of the receptors to the surface [14].

## 4. Unique Expression Pattern

NMDAR subunits have distinct expression patterns [26] that tightly regulate the development of synapses and drive synaptic plasticity to refine the neuronal network. GluN3A is no exception and perhaps is an influential mediator in shaping synaptic connections. It has a unique developmental expression pattern within several brain regions [55]. Initially expressed in the thalamus, entorhinal cortex, subiculum and several layers of the neocortex, its expression intensifies during the first postnatal weeks in the CA1 field of the hippocampus and in the thalamus. It has also been detected at varying degrees in the spinal cord, medulla, pons, tegmentum and hypothalamus [15, 16, 56]. Its endogenous natural expression pattern has been thoroughly described in rodent, human and macaques [15, 57–59]. Detected as early as embryonic day 15, its expression increases in the first two weeks of postnatal development and thereafter sharply declines from P16 and remains low in adulthood [15, 16, 38, 55]. The retina, olfactory tract, amygdala, and some regions of the cortex do retain low levels of GluN3A into adulthood [58, 60], but its functions remain unknown. This is suggesting that GluN3A could control NMDAR function in a time-dependent manner during critical periods of development. In recent years, studies have confirmed GluN3A expression postsynaptic sites within the PSD but situated at the periphery, at perisynaptic, extrasynaptic, and presynaptic sites, as well as on astrocytes [14, 25, 61] (its functions on astrocytes will be discussed later in Section 8). GluN3B has different spatial and temporal expression patterns and can act independently from GluN3A [42, 49, 50, 62]. GluN3B mRNA expression levels are elevated through development and maintained into adulthood, with a distribution in the pons, midbrain, medulla and spinal cord [42]. Therefore it appears that GluN3A and GluN3B have distinctly different roles in the brain during development and adulthood.

## 5. Which Intracellular Proteins Interact with GluN3A?

The CTD of GluN3A is different from that of GluN1 or GluN2. Its dominant-negative properties on receptor kinetics could have downstream effects on intracellular signaling pathways, protein translation and cytoskeletal protein arrangement. One protein that directly interacts with GluN3A is Protein Phosphatase 2A (PP2A) [63, 64]. PP2A is one of the major serine-threonine phosphatases existing as a heterotrimeric enzyme complex in neurons [65]. This interaction between PP2A and GluN3A drives an increase in the activity levels of the enzyme [64]. This tight interaction is abolished upon NMDAR stimulation, causing a dissociation of PP2A from GluN3A and resulting in the dephosphorylation of Ser 897 on the GluN1 subunit [64]. As PP2A has been implicated in LTD, this interaction between PP2A and GluN3A could implicate the level of LTD by maintaining a high level of activity of PP2A. However, the effects of overexpressing GluN3A in transgenic mice showed no changes in LTD [18]. Interestingly, PP2A expression, like GluN3A, is developmentally regulated, peaking around P8 and declining from P12 to a low level in adulthood.

GluN3A also interacts with MAP1B [66] and MAP1-s [67]. MAP1 family proteins are important in the development of axons and dendrites [68]. By binding to the microtubule lattice, they could drive the trafficking of GluN3A-containing NMDAR to peri- and extrasynaptic sites. However, their binding site on the CTD of GluN3A overlaps with PP2A binding site [63], suggesting that there could be a potential competition or a reciprocal binding pattern of the two to GluN3A. MAP1 proteins could traffic GluN3A to its synaptic location and once it dissociates, PP2A can bind to exert its effects. Future experiments are needed to confirm the role of these interactions.

A third report also demonstrates the interaction of plectrin, CARP-1 (cell cycle and apoptosis regulatory protein 1, a perinuclear phosphoprotein [69]) and GPS2 (G protein suppressor 2) with GluN3A, but the exact role these three proteins exert on GluN3A function is still unknown [70]. As plectrin is a large scaffolding protein that binds to several cytoskeletal proteins, it may play a role in the distribution, localization and clustering of GluN3A to appropriate synaptic sites. Although experimental evidence is lacking, we could suggest GPS2 to be linked with NMDAR activation through the suppression of RAS/MAPK-mediated signaling. CARP-1 function in the CNS has never been confirmed outside the CNS and it has been linked to breast carcinoma cells as a protein increasing apoptosis [70]. Again, further experiments will clarify the precise role of these interactions.

Interestingly, GluN3A interacts with the small GTPase Ras homologue enriched in brain (Rheb). This member of the Ras superfamily of GTP-binding proteins stimulates the activity of the mTOR signaling complex 1 (mTORC1), resulting in protein synthesis [71]. This pathway is heavily involved in the fragile X syndrome, in which the absence of the fragile X mental retardation protein (FMRP) causes cognitive deficits in humans [72, 73]. FMRP activity is dependent on its phosphorylation, under the bidirectional control of ribosomal protein S6 kinases and PP2A, in which the activity of both proteins are modulated by the mTOR pathway [74, 75]. It appears that Rheb interacts to the same region of the CTD of GluN3A [73], as PP2A [63, 64]. It is possible that GluN3A could sequester synaptic Rheb, and therefore act as a break on mTOR activity. Consequently, overexpressing GluN3A could prevent the activation of mTOR, and in contrast silencing GluN3A would promote the activity of mTOR. This could have important regulatory consequences on spine and synapse dynamics.

### 5.1. Intracellular Proteins Targeting GluN3A Endocytosis

GluN3A expression sharply declines after P16 in rodents. Its endocytosis is therefore tightly regulated. A report by Pérez-Otaño et al., [24] in 2006 was the first to highlight the activity dependent interaction of PACSIN1/syndapin, an endocytotic adaptor protein, with GluN3A [24]. More recently, identification of a specific motif within the CTD of GluN3A has also been linked to the endocytotic complex [76].

PACSIN1 is a brain-derived protein involved in synaptic vesicle endocytosis. The conserved Src homology 3 (SH3) domain on PACSIN1 enables protein-protein interactions with endocytotic proteins such as N-WASP and dynamin [77, 78]. The presence of a coiled-coil alpha helical domain on PACSIN1 allows for homodimeric and homotetrameric interactions, this provides multiple SH3 domains that can bind with several endocytotic proteins simultaneously [79, 80]. Activity drives the interaction of PACSIN1 with the CTD of GluN3A, promoting its internalization [24]. Disrupting PACSIN1 expression consequently disrupts the surface expression levels of GluN3A. PACSIN1 phosphorylation has also been linked to Rac1 activation, affecting spine formation [81]. This could occur when Rheb and PP2A are dissociating from GluN3A, exposing the CTD for PACSIN1, leading to activation of the mTOR and Rac1 pathway. PACSIN1 offers a mechanistic way to disrupt GluN3A surface expression.

Another unique portion of the CTD is a conserved YWL motif. Src phosphorylates the tyrosine Y971 residue, promoting the interaction with the *μ*2 subunit of AP2 (activating protein 2, involved in clathrin-mediated endocytosis), recruiting the endocytotic machinery and consequently triggering the removal of GluN3A from the surface [76]. By mutating this YWL motif, endocytosis of GluN3A is dramatically prevented, enhancing the surface expression of GluN3A. In contrast, stimulation of endogenous Src via PACAP-38 (pituitary adenylate cyclase activating peptide shown to activate Src in CA1 cells [76, 82]) promotes the internalization of GluN3A and an overall decrease in surface expression [76]. This motif does not conform to previously identified tyrosine-based endocytotic motifs, usually comprising YXXØ (where X is any amino acid and Ø a bulky hydrophobic residue [76]) and dileucine motifs that bind to AP2. GluN2B contains such a motif and phosphorylation of the tyrosine residue by Fyn inhibits AP2 binding and prevents GluN2B internalization [83]. Conversely phosphorylation by Src also inhibits the endocytosis of GluN2A, thus suggesting that tyrosine phosphorylation drives the removal of GluN3A while maintaining the surface expression of GluN2 subunits. What remains unclear is that if PACSIN1 and the phosphorylation of the YWL motif act as two independent systems or if they work in conjunction with one another to assist the removal of GluN3A.

## 6. GluN3A Effects on Dendritic Spine Dynamics

Dendritic spines are very dynamic structures that undergo a continuous process of formation and elimination that is particularly active during development [84]. These mechanisms are also modulated by neuronal activity and notably regulated in an NMDAR-dependent manner [85–87]. An implication of GluN3A in these mechanisms was not unexpected as GluN3A expression peaks at a time when this structural plasticity is most distinguished.

Indeed, in the wake of GluN3A discovery, Das et al., 1998 [17] reported that mice lacking GluN3A showed an increase in spine density in cortical neurons at P19, with a tendency for spine heads to be enlarged and elongated [17]. This coincided with an enhancement of NMDAR responses and an absence of the smaller conductance state seen in GluN3A positive neurons. This was the first evidence to suggest that the absence of GluN3A during its endogenous expression window can affect spine dynamics.

In contrast, ten years after this initial paper Roberts et al., 2009 [18] showed in a transgenic mouse model overexpressing GluN3A (beyond its natural time window), that spine density is reduced. This reduction mostly concerns mature mushroom-shaped spines that also exhibit slightly smaller PSD length [18].

A detailed review by Henson et al. in 2010 [41] proposed two hypotheses to account for these observations: the “synaptic brake hypothesis” and the “synaptic elimination hypothesis.” However, these two hypotheses are still to be explored. The synaptic brake hypothesis suggests that GluN3A-containing receptors limit synapse formation, and its dominant-negative mechanisms on current and Ca^2+^ influx prevent synapse plasticity [41]. Only their time- and activity dependent removal will then allow conventional NMDAR to drive the maturation of the synapse. This interplay between GluN3A-containing receptors and mature GluN1/GluN2-containing receptors will firstly prevent early maturation of synapses, and secondly only strengthen and stabilize the appropriate synapses, leading to a properly formed neuronal circuitry [41].

On the contrary, the synaptic elimination hypothesis proposes that GluN3A-containing receptors act as a tagging mechanism to label weak and inactive synapses, which will promote the retraction, and final elimination of the spine and synapse [41]. Those spines receiving sufficient activity will therefore drive the removal of GluN3A via its internalization mechanisms and drive the insertion of mature NMDAR.

These two hypotheses are still open and future experiments should determine (i) whether GluN3A does in fact prevent spine formation, or (ii) whether expression of GluN3A promotes spine instability and elimination, and prevents plasticity at individual spines. Additionally, the actual downstream mechanisms underlying GluN3A influence on spine formation or elimination still remain to be identified.

Understanding the influence of GluN3A on spine dynamics could provide important new insights on its implications in neurodegenerative diseases.

## 7. GluN3A in Disease

Abnormalities in dendritic spine density, turnover, formation and elimination have been implicated in disorders from mental retardation to Huntington's disease (HD) and Alzheimer's disease [88]. Furthermore, NMDAR hyperactivity or hypoactivity are associated with several neurological conditions such as Alzheimer's disease, Parkinson's disease, schizophrenia, depression and ischemia [26, 89–91]. Growing evidence in recent years has implicated GluN3A in various disorders of the CNS. With respect to NMDAR hypofunction and dendritic spine abnormalities, see reviews [40, 41]. In the past year, GluN3A has been directly implicated in HD and in cocaine addiction, furthermore there are additional reports confirming GluN3A neuroprotective properties in ischemia and in striatal lesions.

HD is a debilitating neurodegenerative disease in which patients carry a mutation in the Huntingtin (mHtt) protein [92, 93]. An expansion of the polyglutamine chain in mHtt forms aggregates leading to synaptic failure and neuronal death, predominately in medium spiny neurons (MSN) of the striatum [94, 95]. Htt has numerous binding partners associated with roles in transcriptional regulation, intracellular trafficking and cytoskeletal organization [96]. PACSIN1 is one such protein, which has a high affinity to interact with mHtt, the longer the polyQ chain the stronger the interaction [97]. This interaction sequesters PACSIN1 from its usual synaptic location. The redistribution of PACSIN1 promotes the reinsertion of GluN3A-containing NMDAR at the synaptic plasma membrane in HD mouse models [97]. This is consistent with analyses of postmortem tissue from human HD patients, in which there is an increase in GluN3A levels [97]. Motor and cognitive deficits as well as decreases in spine density and striatal atrophy of MSNs are rescued in HD mouse models lacking GluN3A [97]. This can provide options to target either GluN3A or PACSIN1 as a therapy in early stages of HD.

An interesting new report has also linked GluN3A with altered NMDAR transmission in cocaine-induced addiction. Cocaine exposure drives a redistribution of AMPAR and NMDAR on dopamine neurons in the ventral tegmental area [98–100]. Emphasis in recent years has focused on the AMPAR switch from Ca^2+^-impermeable GluA2-containing receptors to Ca^2+^-permeable GluA2-lacking receptors induced by a single injection of cocaine [101]. This also coincides with an increase in AMPAR/NMDAR ratio, caused by enlarged AMPAR-mediated EPSC amplitudes together with reduced amplitudes of NMDAR-EPSCs [100]. The reduction of NMDAR-EPSCs amplitude is the result of the reinsertion of GluN2B as well as GluN3A-containing NMDAR [102], this accounts for the reduction in outward rectification and alteration in Mg^2+^ block. Indeed, GluN3A KO mice lack this effect. In addition, DA neurons from these mice and from neurons transfected with a shRNA for GluN3A failed to exhibit cocaine-evoked plasticity of NMDA and AMPA receptors [102]. Furthermore, it was found that NMDAR transmission could be reestablished by changing the ratio of GluN1/2B/3A to GluN1/2A through activation of mGluR1 [102]. This offers prospects for targeting either GluN3A or mGluR1 receptors to restore normal synaptic transmission in drug-addictive behaviour.

As previously discussed, GluN3A could implicate some neuroprotective properties [41, 103]. Excitotoxicity that occurs in disease states is often the result of an overactivation of Ca^2+^-permeable NMDAR. The dominant-negative effects of GluN3A on Ca^2+^ permeability could be of interest to reduce Ca^2+^ influx and consequently prevent cell death.

Indeed, in transgenic GluN3A overexpressing mice model, striatal MSN-induced death via the neurotoxin 3-nitropropionic acid (3-NP) was significantly prevented [104]. These mice also show less dystonia and an improvement in hindlimb clasping and locomotor ability. Synaptic versus extrasynaptic NMDAR promote different survival or cell death pathways, respectively [26]. Synaptic GluN2A-containing NMDAR predominately protect against cell death [105]. However, extrasynaptic GluN2B-containing NMDAR appear to promote cell death pathways [106]. This study shows a tendency for the formation of GluN1/2B/3A-containing receptors to be located at extrasynaptic sites, and the presence of GluN3A prevents cell death signaling. This is further characterised by a decrease in the activation of calpains, proteases that cleave fodrin, and striatal-enriched protein tyrosine phosphatases that trigger major cell death signaling pathways [104]. However, GluN3A cannot account for complete neuroprotection as only 52% of cells survived in this mouse model [104]. Ischemia and hypoxia induce an endogenous upregulation of GluN3A in rat hippocampal and prefrontal neurons [107], further supporting the hypothesis that GluN3A-containing NMDAR have neuroprotective properties. Expression of GluN3A reduces the Ca^2+^ influx as well as hydroxyl radicals and nitric oxide levels after glutamate insult.

These studies provide evidence that GluN3A can act in a neuroprotective manner [103]. This subunit that is usually expressed only during development, appears to be upregulated in response to toxic insult induced by excessive glutamate activation of NMDAR. Perhaps the therapeutic benefit of this could be to target a quick and efficient increase in GluN3A expression that can alleviate the neuronal death by preventing Ca^2+^ influx and cell death.

## 8. Perspectives: GluN3A and Astrocytes

In addition to neurons, GluN3A has also been reported to be expressed in astrocytes and could consequently participate in their bidirectional communication. Astrocytes are the most important glial cell type interacting with neurons, especially at glutamatergic synapses [20]. Astrocytes send fine cellular processes in the vicinity of the synapses. These so-called perisynaptic astrocytic processes express glutamate receptors and transporters important for the bidirectional communication with neurons [108]. In particular, they express mGluR [109, 110] that are mainly responsible for the astrocyte-to-neuron transmission [111]. mGluR activation following synaptic release of glutamate triggers intracellular Ca^2+^ elevation in astrocytes [111, 112] which in turn can induce the release of several transmitters from astrocytes (e.g., gliotransmitters) [20]. Among these gliotransmitters glutamate [113] and D-serine [114] have been identified and both of them can bind to neuronal iGluR including NMDAR [22, 23] giving rise to the concept of a tripartite synapse [115]. The effects of gliotransmitters probably occur in the periphery of the synapse on extrasynaptic receptors [22, 111]. This fits with the localization of GluN3A which is primarily expressed at extrasynaptic sites [24]. These extrasynaptic GluN3A-containing receptors could therefore participate in the mechanisms of gliotransmission.

In addition to mGluR, astrocytes also express iGluR. The presence of AMPA and Kainate receptors on astrocytes have already been described [116]. More recently, NMDAR have been detected on astrocytes as well [117]. However, the exact role of iGluR on astrocytes remains unclear, although we can suggest their participation in neuron-to-glia communication. Glutamate application onto astrocytes evokes three types of responses: an AMPAR, an NMDAR and a glutamate transporter response [118]. Furthermore, axonal stimulations in cortical layer IV–VI give rise to inward currents in astrocytes in layer II, demonstrating that iGluR are involved in neuronal-to-astrocyte communication [118]. All seven subunits of NMDAR were confirmed to be expressed at varying levels in both fetal and adult human astrocytes [25].

Supporting the notion that mGluR mediates a majority of glutamatergic Ca^2+^ signaling in cortical astrocytes [111, 119], activation of NMDAR can facilitate a rise in intracellular Ca^2+^ in astrocytes [25, 120]. However, Palygin et al. in 2011 [120] reported that these NMDAR on astrocytes display lower Ca^2+^ permeability and weak Mg^2+^ block, suggesting the presence of a GluN3 subunit. Thus, the stoichiometry of these NMDAR could contain GluN1/GluN2C or 2D and GluN3A subunits. The application of D-serine or glycine evoked only small responses, while the responses to NMDA and D-serine are large, this outrules the presence of excitatory glycine GluN1/3 receptors and suggests a heterotrimeric composition of astrocytic NMDAR [120].

Why would GluN3A-containing NMDAR be expressed on astrocytes? One important property of GluN3A-containing NMDAR is their lack of Mg^2+^ sensitivity. As astrocytes and astrocytic processes are hyperpolarized, it therefore might be important to express NMDAR that are not fully blocked under resting conditions. In addition, as mentioned above, Ca^2+^ excitability of astrocytes is pivotal for their interaction with neurons and NMDAR are known to potentiate Ca^2+^ responses in astrocytes [111]. It is generally accepted that astrocytic Ca^2+^ signals evoked by glutamatergic activity are mainly mediated by mGluRs. However, mGluR expression in astrocytes decreases during development [110] as does GluN3A. It might be plausible that GluN3A exert an attenuating effect on Ca^2+^ when mGluR are high and inversely modulate astrocytic Ca^2+^ signals through development.

Furthermore, perisynaptic astrocytic processes appear to be highly plastic structures, see [21, 121]. This form of structural plasticity suggests astrocytes to be active players in the mechanisms of synapse formation, stabilization [122, 123] and maturation [124]. Interestingly, NMDAR are important in this process [123], as well as in the increased synaptic coverage by perisynaptic astrocytic processes observed after LTP [125]. In fact, the Rac1 pathway in hippocampal astrocytes [122] as well as in Bergman glia (astrocytes of the cerebellum) [126] have been identified as the mechanism driving astrocytic movements [127]. Rac1 is in turn well known to regulate spine dynamics [128, 129]. As described above, Rac1 could be indirectly linked with GluN3A via PACSIN1, suggesting a possible role for astrocytic GluN3A in mediating effects of synaptic structure, and function through the dynamics of astrocytic processes.

## 9. Discussion

**Figure 2:**
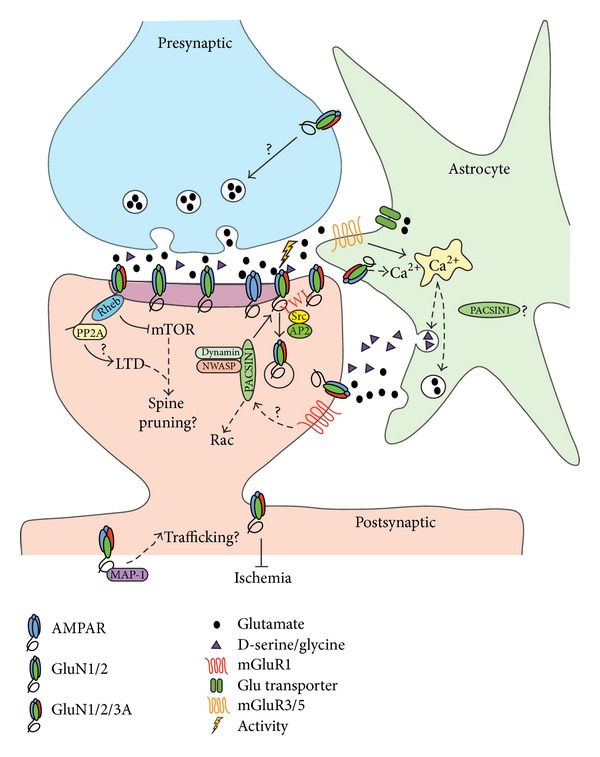
Schematic of the proposed distribution of GluN3A-containing NMDAR in the pre- and postsynaptic sites as well as on astrocytic processes. Endocytosis of GluN3A is activity dependent, driving PACSIN1 binding and Src phosphorylation of the YWL motif to selectively internalize GluN3A-containing NMDAR. Other molecular interactions with GluN3A could play roles in trafficking of the receptor subunit and dendritic spine dynamics. The roles of GluN3A in astrocytic processes still remain unclear but it could potentially exert an attenuating effect on calcium entry in a reciprocal manner to mGluR calcium entry.

Our understanding of the role of GluN3A in neurons, in astrocytes, and of its role in the tripartite synapse in general is slowly emerging (Figure 2).

What does the future hold for GluN3A? Pharmacological agents that specifically act on GluN3A do not yet exist. To achieve this, more research would be needed to confirm the precise stoichiometry of GluN3A-containing NMDAR in neurons and astrocytes. This is a difficult issue as the stoichiometry can be dependent on splice variants. Furthermore, GluN1 has eight functionally distinct splice variants in total, in which GluN1-1a appears to easily coassemble with GluN3A [14]. In fact, there is an additional splice variant of GluN3A, a longer version that consists of additional 60 amino acids in the CTD and is only detected at present in rodent [56]. The precise role for this variant is unknown.

Gathering further information on intracellular proteins that interact with GluN3A could also be beneficial in terms of our understanding of plasticity and dendritic spine dynamics. The most encouraging results to date are the interactions with PP2A and Rheb, both of which can have significant downstream effects, notably regarding LTD or LTP. Furthermore, while Rheb is bound to GluN3A the mTOR pathway is being suppressed, potentially affecting protein translation and preventing the maturation of the synapse. Further research is required to determine if astrocytic GluN3A drives the same interactions. PACSIN1, which has been implicated in GluN3A activity dependent removal and has a specific role in the pathology of HD, is also expressed in astrocytes [130]. Understanding its function in astrocytes could shed new light on the possible role of GluN3A in gliotransmission. Finally, elucidating the role of GluN3A in spine dynamics could reveal its role in structural and functional plasticity. Enhanced GluN3A expression promotes a decrease in spine density but the actual mechanism responsible for this spine density reduction is not known. As with HD, it appears that the reinsertion of GluN3A can promote spine density reduction even in the adult brain. It would be interesting to understand whether the reactivation or reinsertion of GluN3A into the synapse could reactivate a critical period as seen in early development. Furthermore, it would be important to know whether the expression of GluN3A is able to affect neuron-glia interactions and whether this is modulated during development as already seen with mGluR [110].

Overall, the glutamatergic tripartite synapse concept has an exciting future ahead. Piecing together the role of the ionotropic and metabotropic glutamate receptor families as well as transporters and transmitters will offer potential therapeutic interventions to target many synaptopathies.
